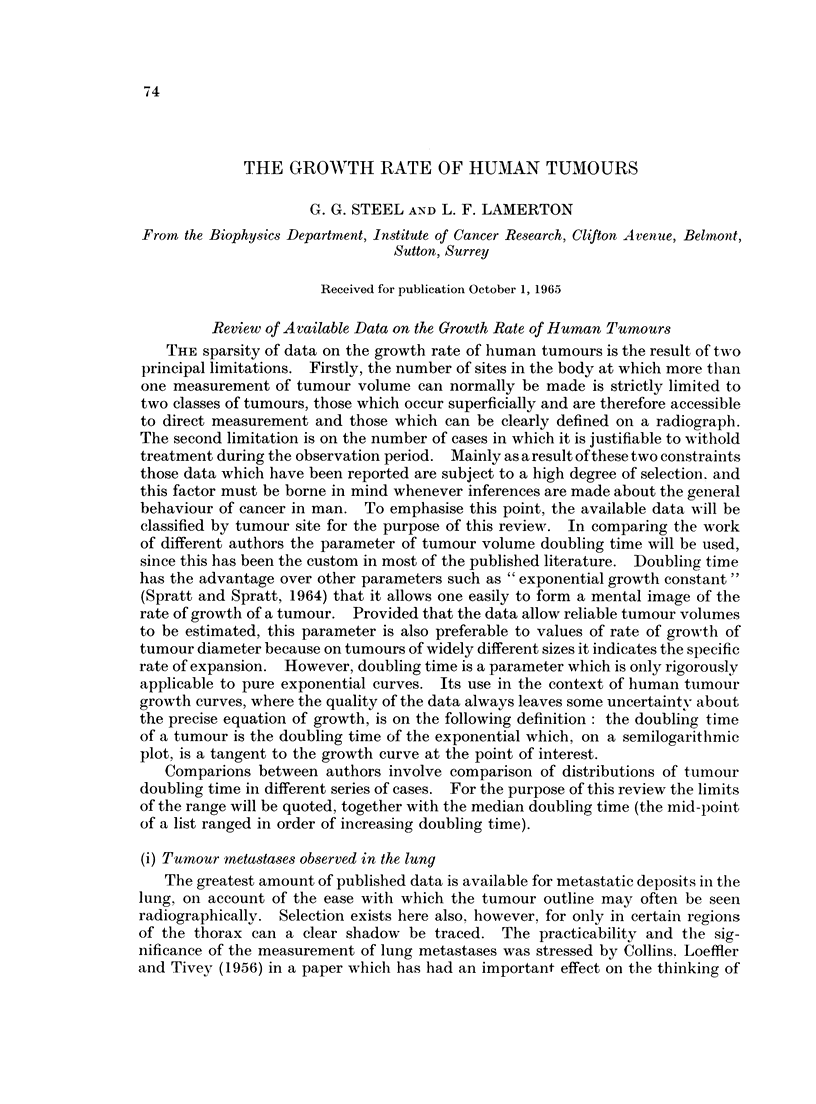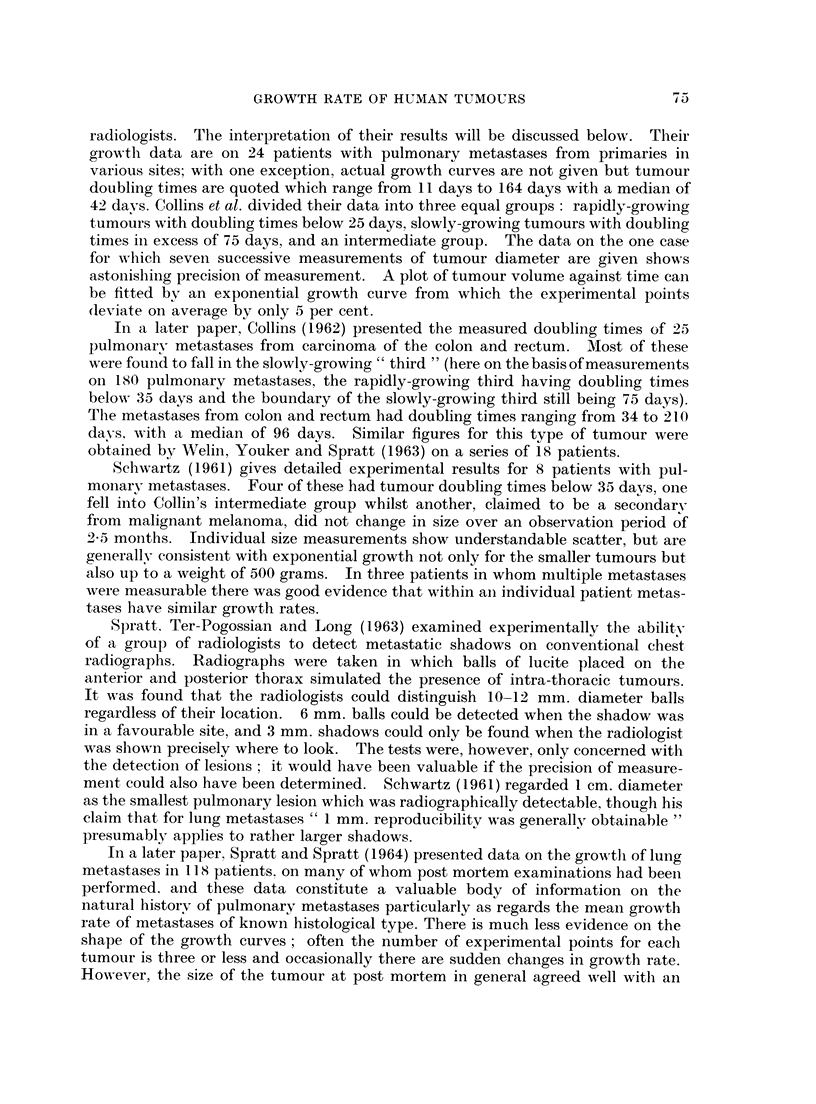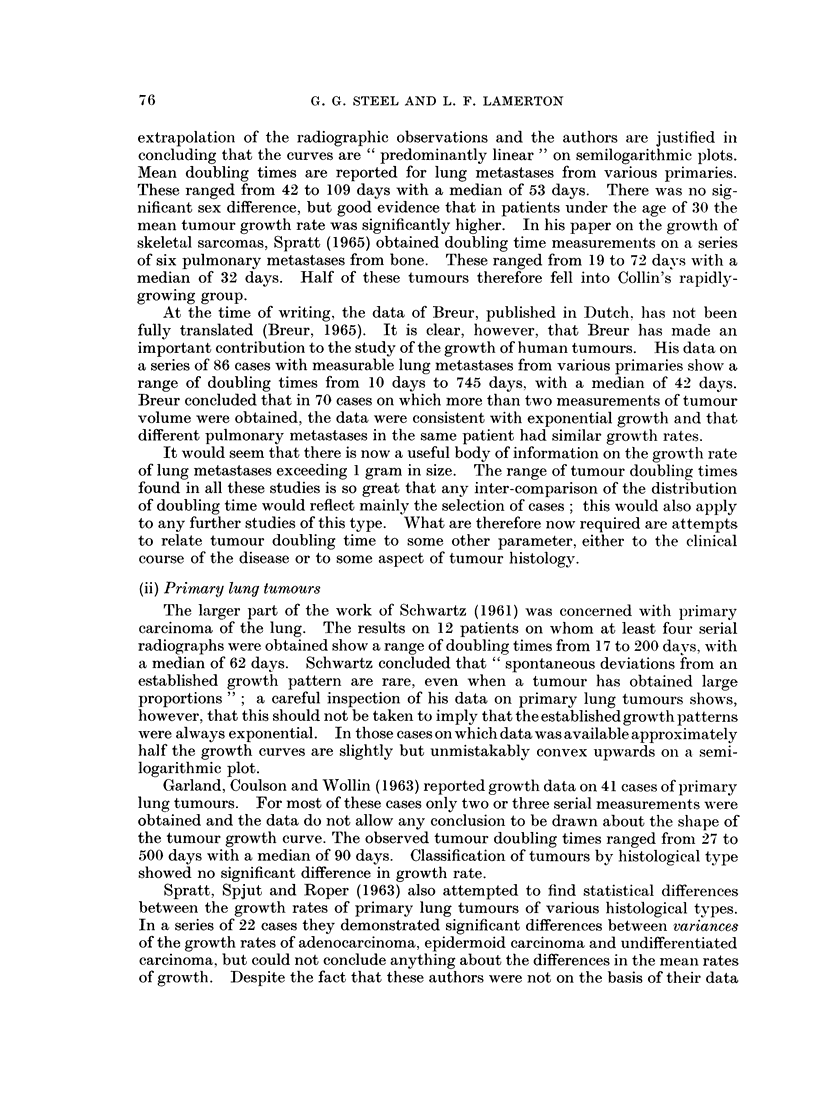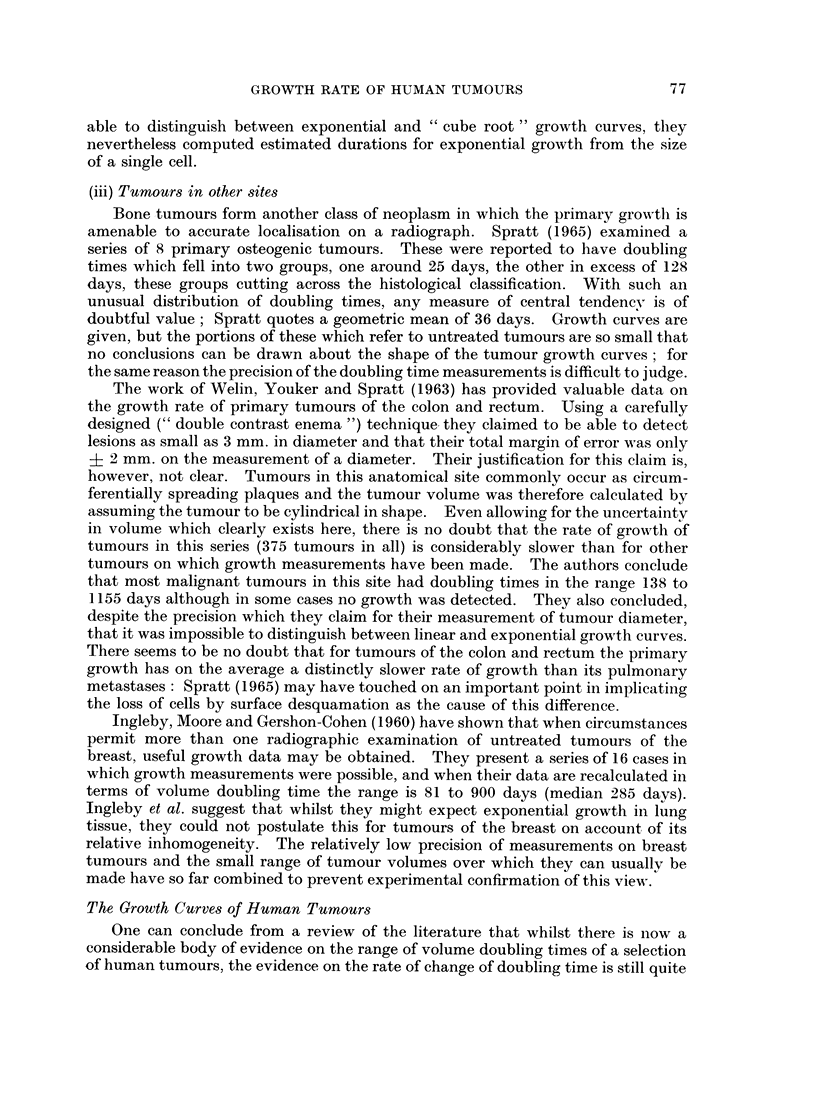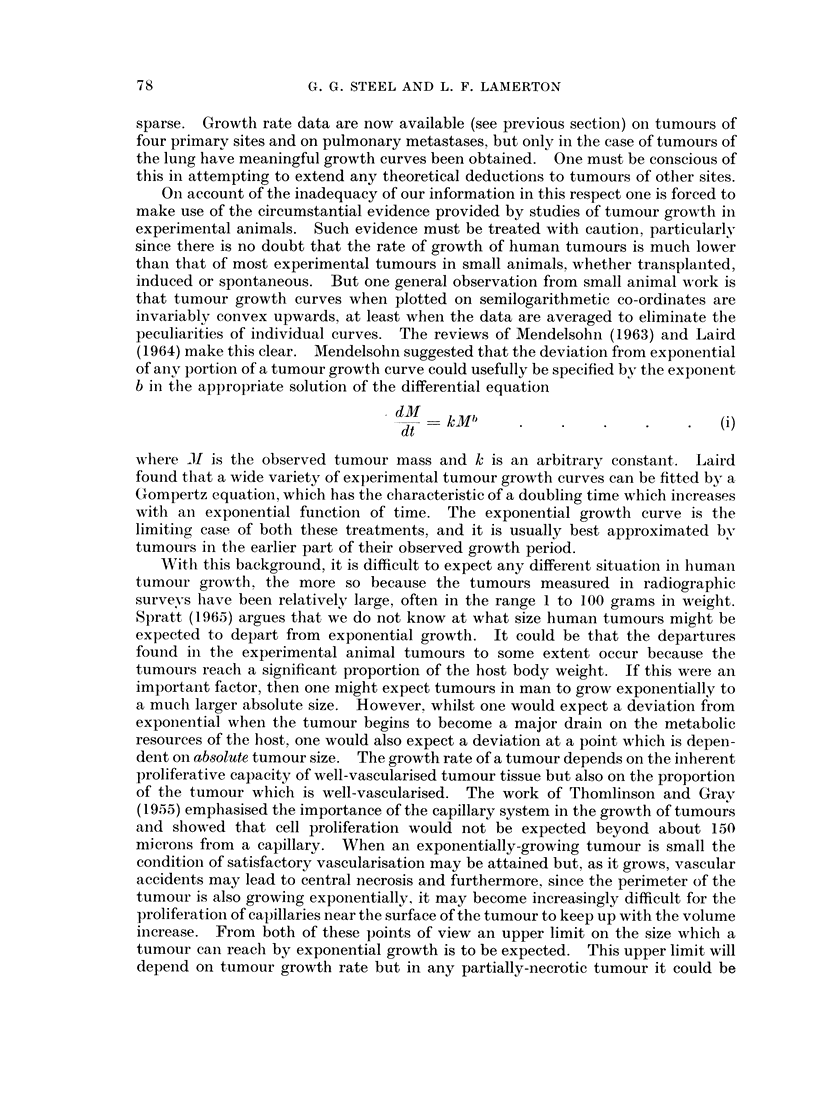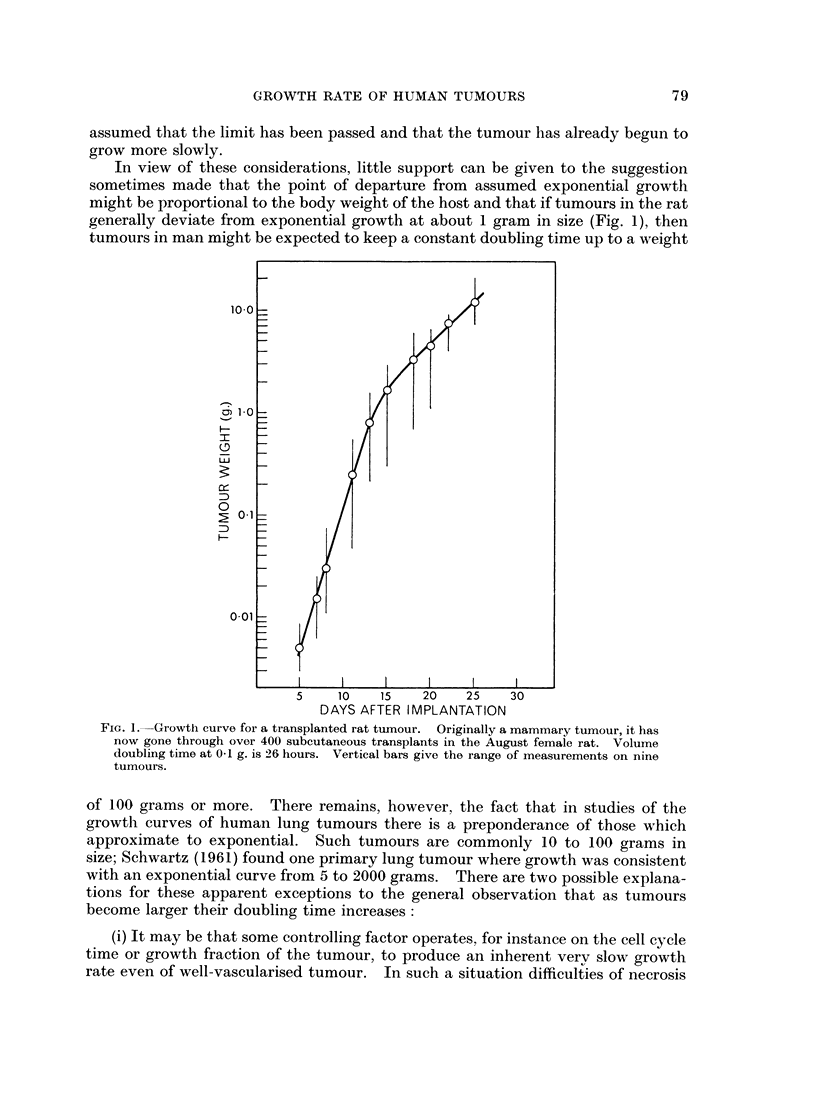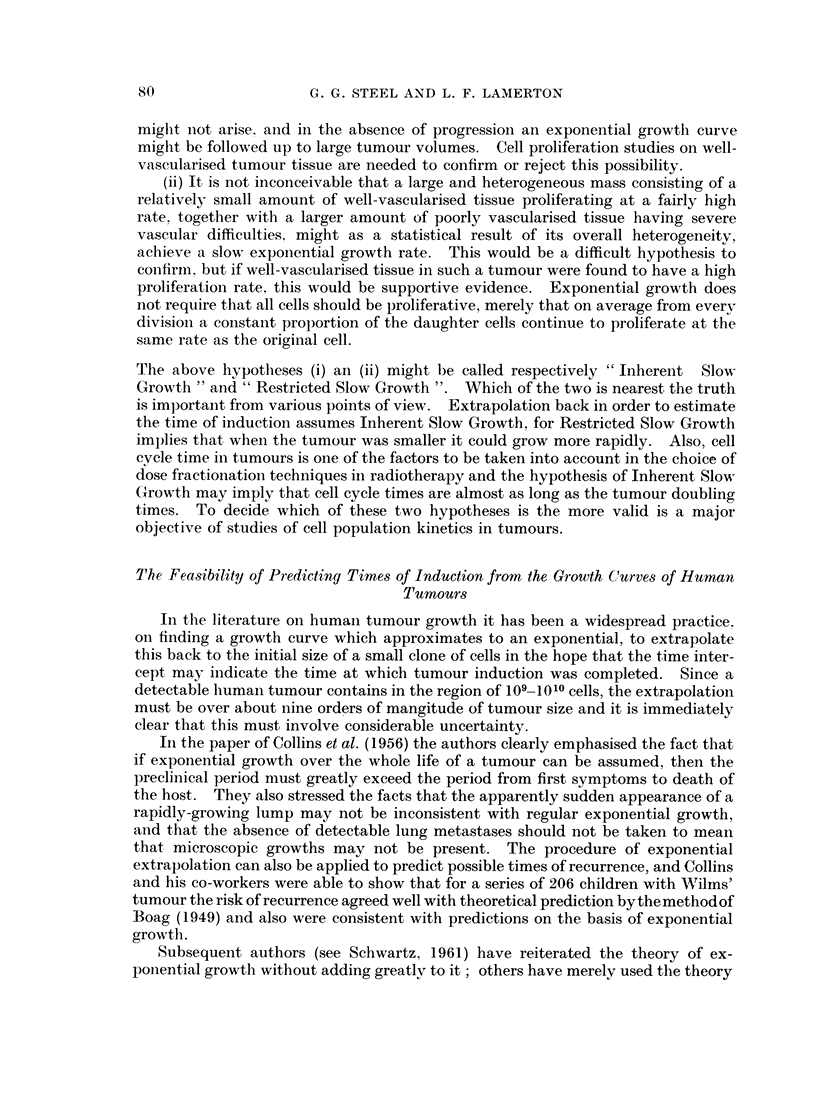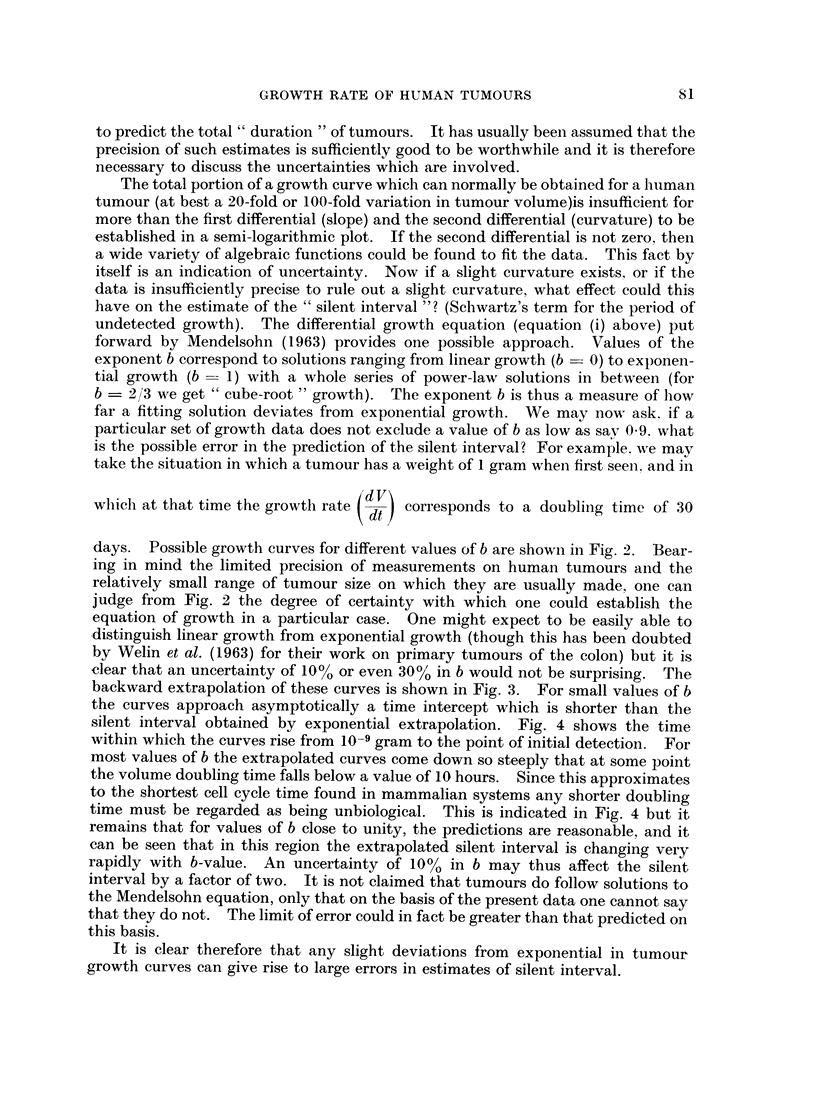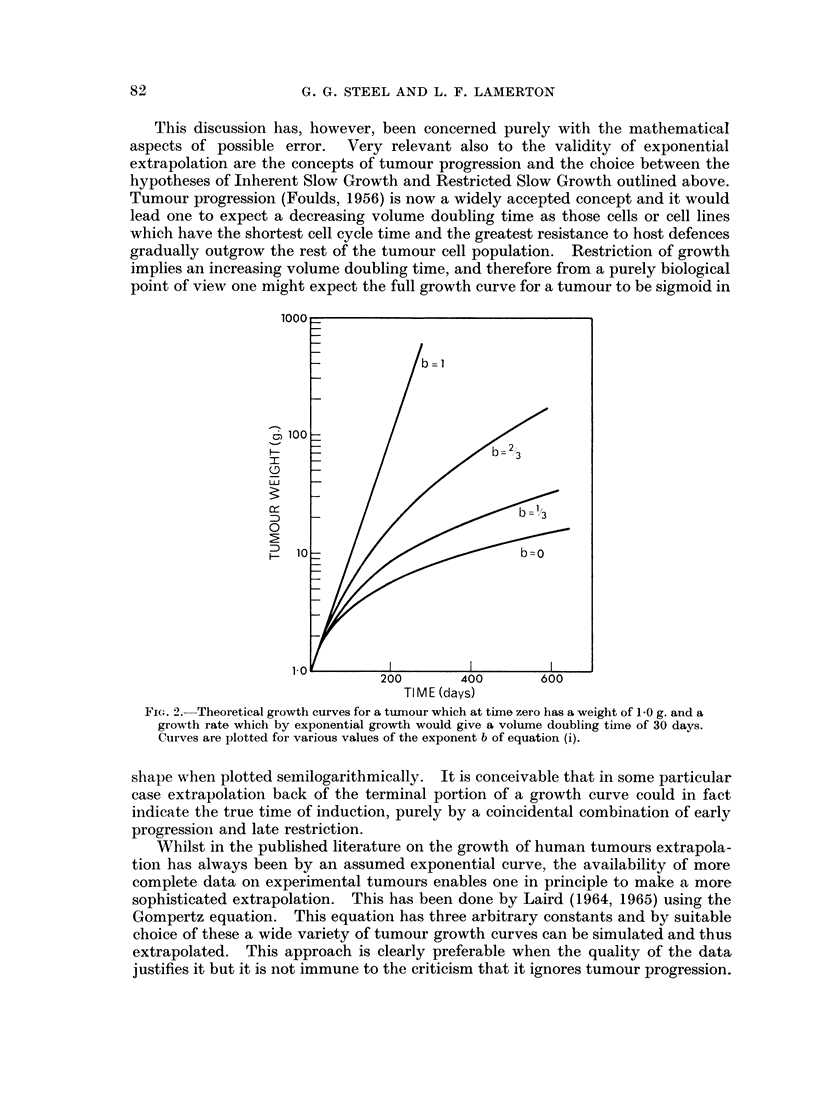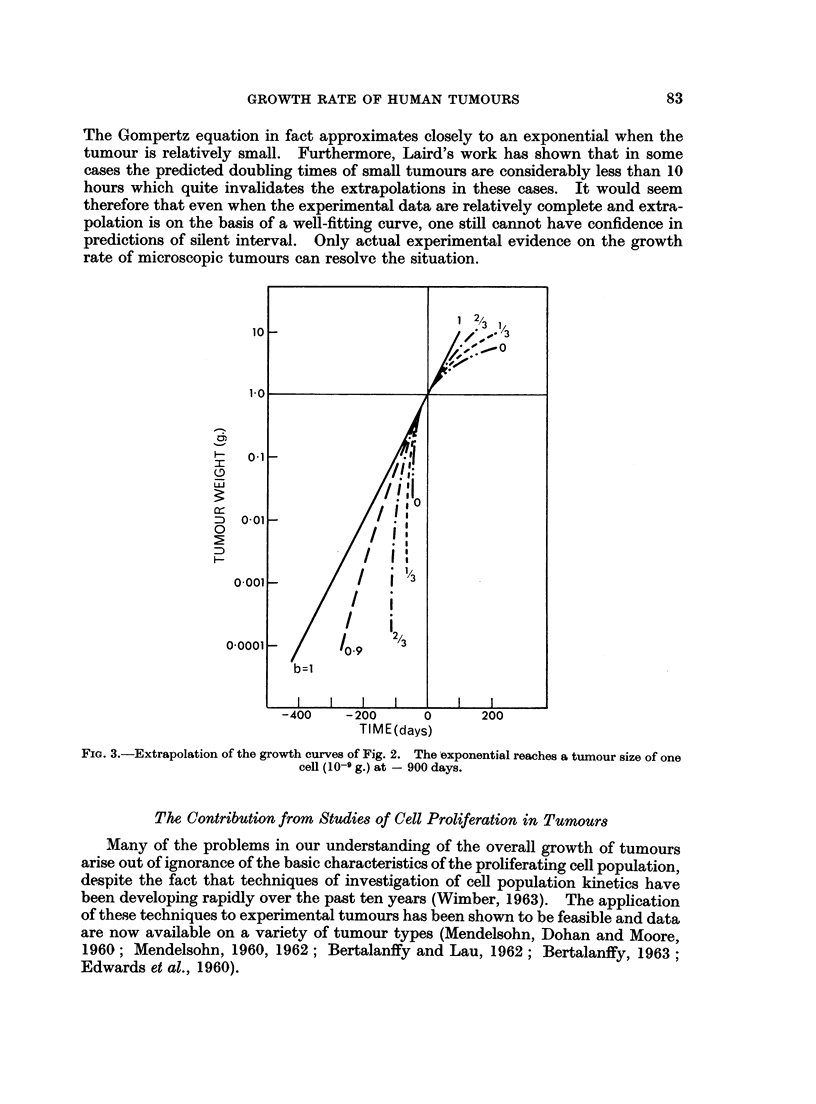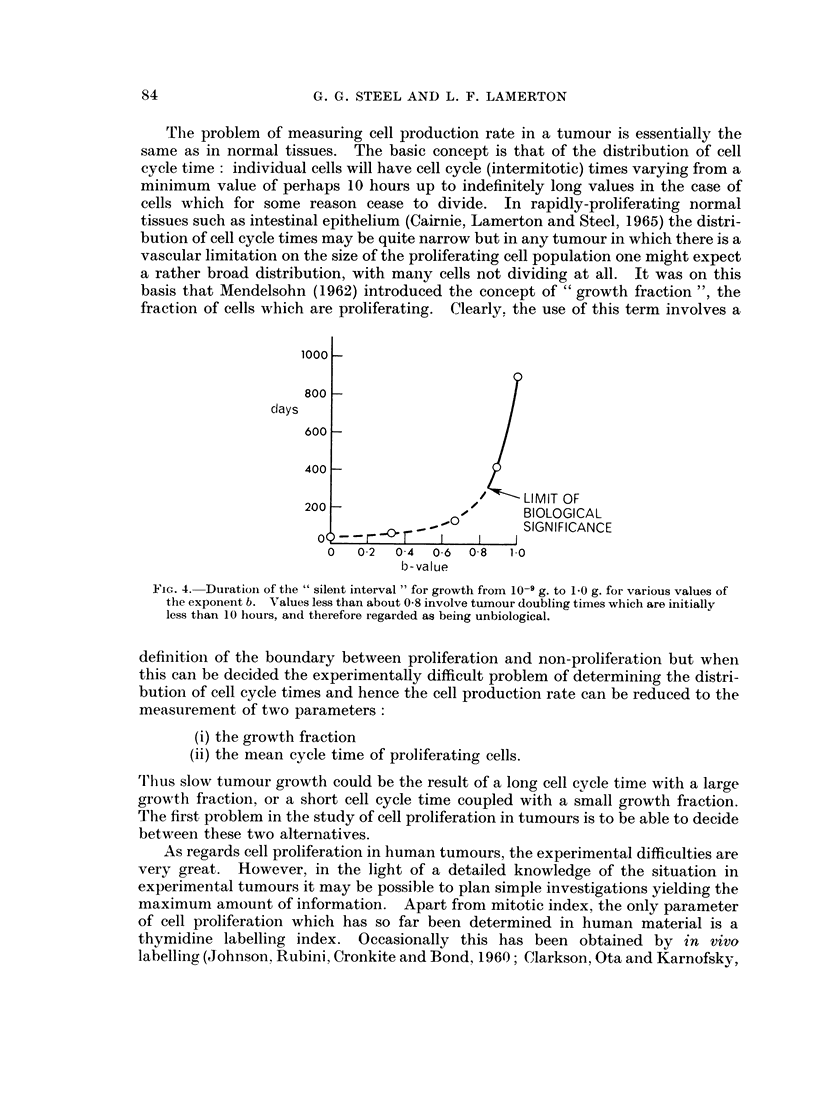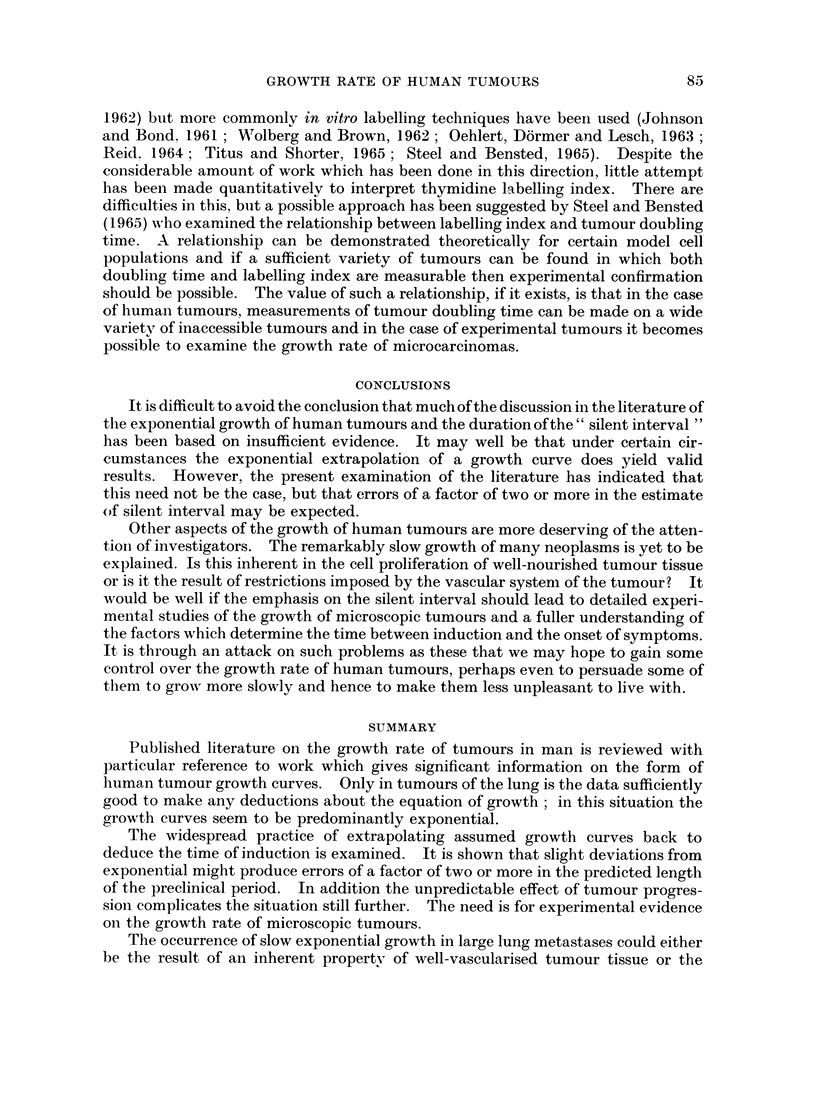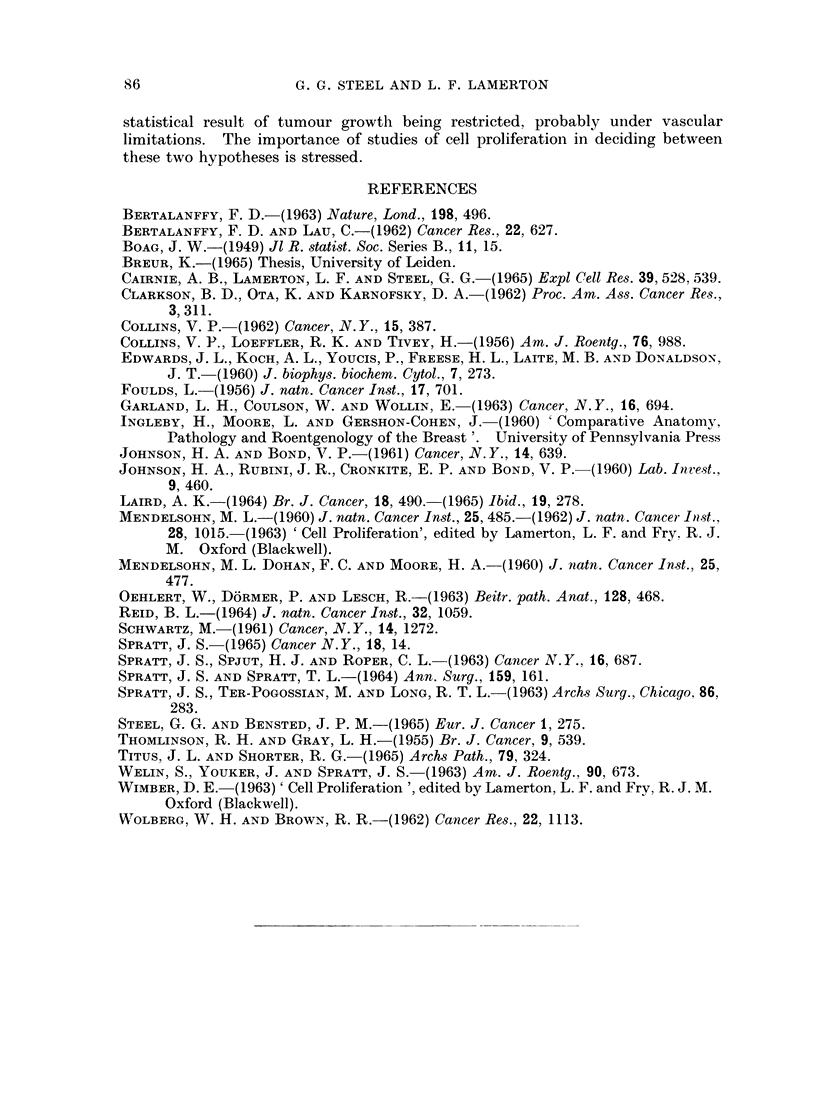# The growth rate of human tumours.

**DOI:** 10.1038/bjc.1966.9

**Published:** 1966-03

**Authors:** G. G. Steel, L. F. Lamerton


### THE GROWTH RATE OF HUMAN TUMOURS

G. G. STEEL AND L. F. LAMERTON

*From the Biophysics Department, Institute of Cancer Research, Clifton Avenue, Belmont, Sutton, Surrey*

Received for publication October 1, 1965

### *Review of Available Data on the Growth Rate of Human Tumours*

THE sparsity of data on the growth rate of human tumours is the result of two principal limitations. Firstly, the number of sites in the body at which more than one measurement of tumour volume can normally be made is strictly limited to two classes of tumours, those which occur superficially and are therefore accessible to direct measurement and those which can be clearly defined on a radiograph. The second limitation is on the number of cases in which it is justifiable to withold treatment during the observation period. Mainly as a result of these two constraints those data which have been reported are subject to a high degree of selection. and this factor must be borne in mind whenever inferences are made about the general behaviour of cancer in man. To emphasise this point, the available data will be classified by tumour site for the purpose of this review. In comparing the work of different authors the parameter of tumour volume doubling time will be used, since this has been the custom in most of the published literature. Doubling time has the advantage over other parameters such as "exponential growth constant" (Spratt and Spratt, 1964) that it allows one easily to form a mental image of the rate of growth of a tumour. Provided that the data allow reliable tumour volumes to be estimated, this parameter is also preferable to values of rate of growth of tumour diameter because on tumours of widely different sizes it indicates the specific rate of expansion. However, doubling time is a parameter which is only rigorously applicable to pure exponential curves. Its use in the context of human tumour growth curves, where the quality of the data always leaves some uncertainty about the precise equation of growth, is on the following definition : the doubling time of a tumour is the doubling time of the exponential which, on a semilogarithmic plot, is a tangent to the growth curve at the point of interest.

Comparions between authors involve comparison of distributions of tumour doubling time in different series of cases. For the purpose of this review the limits of the range will be quoted, together with the median doubling time (the mid-point of a list ranged in order of increasing doubling time).

### (i) *Tumour metastases observed in the lung*

The greatest amount of published data is available for metastatic deposits in the lung, on account of the ease with which the tumour outline may often be seen radiographically. Selection exists here also, however, for only in certain regions of the thorax can a clear shadow be traced. The practicability and the significance of the measurement of lung metastases was stressed by Collins. Loeffler and Tivey (1956) in a paper which has had an important effect on the thinking of radiologists. The interpretation of their results will be discussed below. Their growth data are on 24 patients with pulmonary metastases from primaries in various sites; with one exception, actual growth curves are not given but tumour doubling times are quoted which range from 11 days to 164 days with a median of 42 days. Collins *et al.* divided their data into three equal groups : rapidly-growing tumours with doubling times below 25 days, slowly-growing tumours with doubling times in excess of 75 days, and an intermediate group. The data on the one case for which seven successive measurements of tumour diameter are given shows astonishing precision of measurement. A plot of tumour volume against time can be fitted by an exponential growth curve from which the experimental points deviate on average by only 5 per cent.

In a later paper, Collins (1962) presented the measured doubling times of 25 pulmonary metastases from carcinoma of the colon and rectum. Most of these were found to fall in the slowly-growing " third " (here on the basis of measurements on 180 pulmonary metastases, the rapidly-growing third having doubling times below 35 days and the boundary of the slowly-growing third still being 75 days). The metastases from colon and rectum had doubling times ranging from 34 to 210 days, with a median of 96 days. Similar figures for this type of tumour were obtained by Welin, Youker and Spratt (1963) on a series of 18 patients.

Schwartz (1961) gives detailed experimental results for 8 patients with pulmonary metastases. Four of these had tumour doubling times below 35 days, one fell into Collin's intermediate group whilst another, claimed to be a secondary from malignant melanoma, did not change in size over an observation period of 2·5 months. Individual size measurements show understandable scatter, but are generally consistent with exponential growth not only for the smaller tumours but also up to a weight of 500 grams. In three patients in whom multiple metastases were measurable there was good evidence that within an individual patient metastases have similar growth rates.

Spratt, Ter-Pogossian and Long (1963) examined experimentally the ability of a group of radiologists to detect metastatic shadows on conventional chest radiographs. Radiographs were taken in which balls of lucite placed on the anterior and posterior thorax simulated the presence of intra-thoracic tumours. It was found that the radiologists could distinguish 10–12 mm. diameter balls regardless of their location. 6 mm. balls could be detected when the shadow was in a favourable site, and 3 mm. shadows could only be found when the radiologist was shown precisely where to look. The tests were, however, only concerned with the detection of lesions ; it would have been valuable if the precision of measurement could also have been determined. Schwartz (1961) regarded 1 cm. diameter as the smallest pulmonary lesion which was radiographically detectable, though his claim that for lung metastases " 1 mm. reproducibility was generally obtainable " presumably applies to rather larger shadows.

In a later paper, Spratt and Spratt (1964) presented data on the growth of lung metastases in 118 patients, on many of whom post mortem examinations had been performed, and these data constitute a valuable body of information on the natural history of pulmonary metastases particularly as regards the mean growth rate of metastases of known histological type. There is much less evidence on the shape of the growth curves ; often the number of experimental points for each tumour is three or less and occasionally there are sudden changes in growth rate. However, the size of the tumour at post mortem in general agreed well with an extrapolation of the radiographic observations and the authors are justified in concluding that the curves are " predominantly linear " on semilogarithmic plots. Mean doubling times are reported for lung metastases from various primaries. These ranged from 42 to 109 days with a median of 53 days. There was no significant sex difference, but good evidence that in patients under the age of 30 the mean tumour growth rate was significantly higher. In his paper on the growth of skeletal sarcomas, Spratt (1965) obtained doubling time measurements on a series of six pulmonary metastases from bone. These ranged from 19 to 72 days with a median of 32 days. Half of these tumours therefore fell into Collin's rapidly-growing group.

At the time of writing, the data of Breur, published in Dutch, has not been fully translated (Breur, 1965). It is clear, however, that Breur has made an important contribution to the study of the growth of human tumours. His data on a series of 86 cases with measurable lung metastases from various primaries show a range of doubling times from 10 days to 745 days, with a median of 42 days. Breur concluded that in 70 cases on which more than two measurements of tumour volume were obtained, the data were consistent with exponential growth and that different pulmonary metastases in the same patient had similar growth rates.

It would seem that there is now a useful body of information on the growth rate of lung metastases exceeding 1 gram in size. The range of tumour doubling times found in all these studies is so great that any inter-comparison of the distribution of doubling time would reflect mainly the selection of cases ; this would also apply to any further studies of this type. What are therefore now required are attempts to relate tumour doubling time to some other parameter, either to the clinical course of the disease or to some aspect of tumour histology.

(ii) *Primary lung tumours*

The larger part of the work of Schwartz (1961) was concerned with primary carcinoma of the lung. The results on 12 patients on whom at least four serial radiographs were obtained show a range of doubling times from 17 to 200 days, with a median of 62 days. Schwartz concluded that " spontaneous deviations from an established growth pattern are rare, even when a tumour has obtained large proportions " ; a careful inspection of his data on primary lung tumours shows, however, that this should not be taken to imply that the established growth patterns were always exponential. In those cases on which data was available approximately half the growth curves are slightly but unmistakably convex upwards on a semi-logarithmic plot.

Garland, Coulson and Wollin (1963) reported growth data on 41 cases of primary lung tumours. For most of these cases only two or three serial measurements were obtained and the data do not allow any conclusion to be drawn about the shape of the tumour growth curve. The observed tumour doubling times ranged from 27 to 500 days with a median of 90 days. Classification of tumours by histological type showed no significant difference in growth rate.

Spratt, Spjut and Roper (1963) also attempted to find statistical differences between the growth rates of primary lung tumours of various histological types. In a series of 22 cases they demonstrated significant differences between *variances* of the growth rates of adenocarcinoma, epidermoid carcinoma and undifferentiated carcinoma, but could not conclude anything about the differences in the mean rates of growth. Despite the fact that these authors were not on the basis of their data able to distinguish between exponential and " cube root " growth curves, they nevertheless computed estimated durations for exponential growth from the size of a single cell.

(iii) *Tumours in other sites*

Bone tumours form another class of neoplasm in which the primary growth is amenable to accurate localisation on a radiograph. Spratt (1965) examined a series of 8 primary osteogenic tumours. These were reported to have doubling times which fell into two groups, one around 25 days, the other in excess of 128 days, these groups cutting across the histological classification. With such an unusual distribution of doubling times, any measure of central tendency is of doubtful value ; Spratt quotes a geometric mean of 36 days. Growth curves are given, but the portions of these which refer to untreated tumours are so small that no conclusions can be drawn about the shape of the tumour growth curves ; for the same reason the precision of the doubling time measurements is difficult to judge.

The work of Welin, Youker and Spratt (1963) has provided valuable data on the growth rate of primary tumours of the colon and rectum. Using a carefully designed (" double contrast enema ") technique they claimed to be able to detect lesions as small as 3 mm. in diameter and that their total margin of error was only $\pm$ 2 mm. on the measurement of a diameter. Their justification for this claim is, however, not clear. Tumours in this anatomical site commonly occur as circumferentially spreading plaques and the tumour volume was therefore calculated by assuming the tumour to be cylindrical in shape. Even allowing for the uncertainty in volume which clearly exists here, there is no doubt that the rate of growth of tumours in this series (375 tumours in all) is considerably slower than for other tumours on which growth measurements have been made. The authors conclude that most malignant tumours in this site had doubling times in the range 138 to 1155 days although in some cases no growth was detected. They also concluded, despite the precision which they claim for their measurement of tumour diameter, that it was impossible to distinguish between linear and exponential growth curves. There seems to be no doubt that for tumours of the colon and rectum the primary growth has on the average a distinctly slower rate of growth than its pulmonary metastases : Spratt (1965) may have touched on an important point in implicating the loss of cells by surface desquamation as the cause of this difference.

Ingleby, Moore and Gershon-Cohen (1960) have shown that when circumstances permit more than one radiographic examination of untreated tumours of the breast, useful growth data may be obtained. They present a series of 16 cases in which growth measurements were possible, and when their data are recalculated in terms of volume doubling time the range is 81 to 900 days (median 285 days). Ingleby *et al.* suggest that whilst they might expect exponential growth in lung tissue, they could not postulate this for tumours of the breast on account of its relative inhomogeneity. The relatively low precision of measurements on breast tumours and the small range of tumour volumes over which they can usually be made have so far combined to prevent experimental confirmation of this view.

*The Growth Curves of Human Tumours*

One can conclude from a review of the literature that whilst there is now a considerable body of evidence on the range of volume doubling times of a selection of human tumours, the evidence on the rate of change of doubling time is still quite sparse. Growth rate data are now available (see previous section) on tumours of four primary sites and on pulmonary metastases, but only in the case of tumours of the lung have meaningful growth curves been obtained. One must be conscious of this in attempting to extend any theoretical deductions to tumours of other sites.

On account of the inadequacy of our information in this respect one is forced to make use of the circumstantial evidence provided by studies of tumour growth in experimental animals. Such evidence must be treated with caution, particularly since there is no doubt that the rate of growth of human tumours is much lower than that of most experimental tumours in small animals, whether transplanted, induced or spontaneous. But one general observation from small animal work is that tumour growth curves when plotted on semilogarithmetic co-ordinates are invariably convex upwards, at least when the data are averaged to eliminate the peculiarities of individual curves. The reviews of Mendelsohn (1963) and Laird (1964) make this clear. Mendelsohn suggested that the deviation from exponential of any portion of a tumour growth curve could usefully be specified by the exponent $b$ in the appropriate solution of the differential equation

$$\frac{dM}{dt} = kM^b \quad . \quad . \quad . \quad . \quad . \quad \text{(i)}$$

where $M$ is the observed tumour mass and $k$ is an arbitrary constant. Laird found that a wide variety of experimental tumour growth curves can be fitted by a Gompertz equation, which has the characteristic of a doubling time which increases with an exponential function of time. The exponential growth curve is the limiting case of both these treatments, and it is usually best approximated by tumours in the earlier part of their observed growth period.

With this background, it is difficult to expect any different situation in human tumour growth, the more so because the tumours measured in radiographic surveys have been relatively large, often in the range 1 to 100 grams in weight. Spratt (1965) argues that we do not know at what size human tumours might be expected to depart from exponential growth. It could be that the departures found in the experimental animal tumours to some extent occur because the tumours reach a significant proportion of the host body weight. If this were an important factor, then one might expect tumours in man to grow exponentially to a much larger absolute size. However, whilst one would expect a deviation from exponential when the tumour begins to become a major drain on the metabolic resources of the host, one would also expect a deviation at a point which is dependent on *absolute* tumour size. The growth rate of a tumour depends on the inherent proliferative capacity of well-vascularised tumour tissue but also on the proportion of the tumour which is well-vascularised. The work of Thomlinson and Gray (1955) emphasised the importance of the capillary system in the growth of tumours and showed that cell proliferation would not be expected beyond about 150 microns from a capillary. When an exponentially-growing tumour is small the condition of satisfactory vascularisation may be attained but, as it grows, vascular accidents may lead to central necrosis and furthermore, since the perimeter of the tumour is also growing exponentially, it may become increasingly difficult for the proliferation of capillaries near the surface of the tumour to keep up with the volume increase. From both of these points of view an upper limit on the size which a tumour can reach by exponential growth is to be expected. This upper limit will depend on tumour growth rate but in any partially-necrotic tumour it could be assumed that the limit has been passed and that the tumour has already begun to grow more slowly.

In view of these considerations, little support can be given to the suggestion sometimes made that the point of departure from assumed exponential growth might be proportional to the body weight of the host and that if tumours in the rat generally deviate from exponential growth at about 1 gram in size (Fig. 1), then tumours in man might be expected to keep a constant doubling time up to a weight

FIG. 1.—Growth curve for a transplanted rat tumour. Originally a mammary tumour, it has now gone through over 400 subcutaneous transplants in the August female rat. Volume doubling time at 0·1 g. is 26 hours. Vertical bars give the range of measurements on nine tumours.

of 100 grams or more. There remains, however, the fact that in studies of the growth curves of human lung tumours there is a preponderance of those which approximate to exponential. Such tumours are commonly 10 to 100 grams in size; Schwartz (1961) found one primary lung tumour where growth was consistent with an exponential curve from 5 to 2000 grams. There are two possible explanations for these apparent exceptions to the general observation that as tumours become larger their doubling time increases :

(i) It may be that some controlling factor operates, for instance on the cell cycle time or growth fraction of the tumour, to produce an inherent very slow growth rate even of well-vascularised tumour. In such a situation difficulties of necrosis might not arise, and in the absence of progression an exponential growth curve might be followed up to large tumour volumes. Cell proliferation studies on well-vascularised tumour tissue are needed to confirm or reject this possibility.

(ii) It is not inconceivable that a large and heterogeneous mass consisting of a relatively small amount of well-vascularised tissue proliferating at a fairly high rate, together with a larger amount of poorly vascularised tissue having severe vascular difficulties, might as a statistical result of its overall heterogeneity, achieve a slow exponential growth rate. This would be a difficult hypothesis to confirm, but if well-vascularised tissue in such a tumour were found to have a high proliferation rate, this would be supportive evidence. Exponential growth does not require that all cells should be proliferative, merely that on average from every division a constant proportion of the daughter cells continue to proliferate at the same rate as the original cell.

The above hypotheses (i) an (ii) might be called respectively " Inherent Slow Growth " and " Restricted Slow Growth ". Which of the two is nearest the truth is important from various points of view. Extrapolation back in order to estimate the time of induction assumes Inherent Slow Growth, for Restricted Slow Growth implies that when the tumour was smaller it could grow more rapidly. Also, cell cycle time in tumours is one of the factors to be taken into account in the choice of dose fractionation techniques in radiotherapy and the hypothesis of Inherent Slow Growth may imply that cell cycle times are almost as long as the tumour doubling times. To decide which of these two hypotheses is the more valid is a major objective of studies of cell population kinetics in tumours.

### *The Feasibility of Predicting Times of Induction from the Growth Curves of Human Tumours*

In the literature on human tumour growth it has been a widespread practice, on finding a growth curve which approximates to an exponential, to extrapolate this back to the initial size of a small clone of cells in the hope that the time intercept may indicate the time at which tumour induction was completed. Since a detectable human tumour contains in the region of $10^9$–$10^{10}$ cells, the extrapolation must be over about nine orders of mangitude of tumour size and it is immediately clear that this must involve considerable uncertainty.

In the paper of Collins *et al.* (1956) the authors clearly emphasised the fact that if exponential growth over the whole life of a tumour can be assumed, then the preclinical period must greatly exceed the period from first symptoms to death of the host. They also stressed the facts that the apparently sudden appearance of a rapidly-growing lump may not be inconsistent with regular exponential growth, and that the absence of detectable lung metastases should not be taken to mean that microscopic growths may not be present. The procedure of exponential extrapolation can also be applied to predict possible times of recurrence, and Collins and his co-workers were able to show that for a series of 206 children with Wilms' tumour the risk of recurrence agreed well with theoretical prediction by the method of Boag (1949) and also were consistent with predictions on the basis of exponential growth.

Subsequent authors (see Schwartz, 1961) have reiterated the theory of exponential growth without adding greatly to it ; others have merely used the theory to predict the total " duration " of tumours. It has usually been assumed that the precision of such estimates is sufficiently good to be worthwhile and it is therefore necessary to discuss the uncertainties which are involved.

The total portion of a growth curve which can normally be obtained for a human tumour (at best a 20-fold or 100-fold variation in tumour volume)is insufficient for more than the first differential (slope) and the second differential (curvature) to be established in a semi-logarithmic plot. If the second differential is not zero, then a wide variety of algebraic functions could be found to fit the data. This fact by itself is an indication of uncertainty. Now if a slight curvature exists, or if the data is insufficiently precise to rule out a slight curvature, what effect could this have on the estimate of the " silent interval "? (Schwartz's term for the period of undetected growth). The differential growth equation (equation (i) above) put forward by Mendelsohn (1963) provides one possible approach. Values of the exponent $b$ correspond to solutions ranging from linear growth ($b = 0$) to exponential growth ($b = 1$) with a whole series of power-law solutions in between (for $b = 2/3$ we get " cube-root " growth). The exponent $b$ is thus a measure of how far a fitting solution deviates from exponential growth. We may now ask, if a particular set of growth data does not exclude a value of $b$ as low as say 0·9, what is the possible error in the prediction of the silent interval? For example, we may take the situation in which a tumour has a weight of 1 gram when first seen, and in which at that time the growth rate $\left(\frac{dV}{dt}\right)$ corresponds to a doubling time of 30 days. Possible growth curves for different values of $b$ are shown in Fig. 2. Bearing in mind the limited precision of measurements on human tumours and the relatively small range of tumour size on which they are usually made, one can judge from Fig. 2 the degree of certainty with which one could establish the equation of growth in a particular case. One might expect to be easily able to distinguish linear growth from exponential growth (though this has been doubted by Welin *et al.* (1963) for their work on primary tumours of the colon) but it is clear that an uncertainty of 10% or even 30% in $b$ would not be surprising. The backward extrapolation of these curves is shown in Fig. 3. For small values of $b$ the curves approach asymptotically a time intercept which is shorter than the silent interval obtained by exponential extrapolation. Fig. 4 shows the time within which the curves rise from $10^{-9}$ gram to the point of initial detection. For most values of $b$ the extrapolated curves come down so steeply that at some point the volume doubling time falls below a value of 10 hours. Since this approximates to the shortest cell cycle time found in mammalian systems any shorter doubling time must be regarded as being unbiological. This is indicated in Fig. 4 but it remains that for values of $b$ close to unity, the predictions are reasonable, and it can be seen that in this region the extrapolated silent interval is changing very rapidly with $b$-value. An uncertainty of 10% in $b$ may thus affect the silent interval by a factor of two. It is not claimed that tumours do follow solutions to the Mendelsohn equation, only that on the basis of the present data one cannot say that they do not. The limit of error could in fact be greater than that predicted on this basis.

It is clear therefore that any slight deviations from exponential in tumour growth curves can give rise to large errors in estimates of silent interval.

This discussion has, however, been concerned purely with the mathematical aspects of possible error. Very relevant also to the validity of exponential extrapolation are the concepts of tumour progression and the choice between the hypotheses of Inherent Slow Growth and Restricted Slow Growth outlined above. Tumour progression (Foulds, 1956) is now a widely accepted concept and it would lead one to expect a decreasing volume doubling time as those cells or cell lines which have the shortest cell cycle time and the greatest resistance to host defences gradually outgrow the rest of the tumour cell population. Restriction of growth implies an increasing volume doubling time, and therefore from a purely biological point of view one might expect the full growth curve for a tumour to be sigmoid in

FIG. 2.—Theoretical growth curves for a tumour which at time zero has a weight of 1·0 g. and a growth rate which by exponential growth would give a volume doubling time of 30 days. Curves are plotted for various values of the exponent $b$ of equation (i).

shape when plotted semilogarithmically. It is conceivable that in some particular case extrapolation back of the terminal portion of a growth curve could in fact indicate the true time of induction, purely by a coincidental combination of early progression and late restriction.

Whilst in the published literature on the growth of human tumours extrapolation has always been by an assumed exponential curve, the availability of more complete data on experimental tumours enables one in principle to make a more sophisticated extrapolation. This has been done by Laird (1964, 1965) using the Gompertz equation. This equation has three arbitrary constants and by suitable choice of these a wide variety of tumour growth curves can be simulated and thus extrapolated. This approach is clearly preferable when the quality of the data justifies it but it is not immune to the criticism that it ignores tumour progression.

The Gompertz equation in fact approximates closely to an exponential when the tumour is relatively small. Furthermore, Laird's work has shown that in some cases the predicted doubling times of small tumours are considerably less than 10 hours which quite invalidates the extrapolations in these cases. It would seem therefore that even when the experimental data are relatively complete and extrapolation is on the basis of a well-fitting curve, one still cannot have confidence in predictions of silent interval. Only actual experimental evidence on the growth rate of microscopic tumours can resolve the situation.

FIG. 3.—Extrapolation of the growth curves of Fig. 2. The exponential reaches a tumour size of one cell ($10^{-9}$ g.) at — 900 days.

*The Contribution from Studies of Cell Proliferation in Tumours*

Many of the problems in our understanding of the overall growth of tumours arise out of ignorance of the basic characteristics of the proliferating cell population, despite the fact that techniques of investigation of cell population kinetics have been developing rapidly over the past ten years (Wimber, 1963). The application of these techniques to experimental tumours has been shown to be feasible and data are now available on a variety of tumour types (Mendelsohn, Dohan and Moore, 1960 ; Mendelsohn, 1960, 1962 ; Bertalanffy and Lau, 1962 ; Bertalanffy, 1963 ; Edwards *et al.*, 1960).

The problem of measuring cell production rate in a tumour is essentially the same as in normal tissues. The basic concept is that of the distribution of cell cycle time : individual cells will have cell cycle (intermitotic) times varying from a minimum value of perhaps 10 hours up to indefinitely long values in the case of cells which for some reason cease to divide. In rapidly-proliferating normal tissues such as intestinal epithelium (Cairnie, Lamerton and Steel, 1965) the distribution of cell cycle times may be quite narrow but in any tumour in which there is a vascular limitation on the size of the proliferating cell population one might expect a rather broad distribution, with many cells not dividing at all. It was on this basis that Mendelsohn (1962) introduced the concept of " growth fraction ", the fraction of cells which are proliferating. Clearly, the use of this term involves a

FIG. 4.—Duration of the " silent interval " for growth from $10^{-9}$ g. to 1·0 g. for various values of the exponent *b*. Values less than about 0·8 involve tumour doubling times which are initially less than 10 hours, and therefore regarded as being unbiological.

definition of the boundary between proliferation and non-proliferation but when this can be decided the experimentally difficult problem of determining the distribution of cell cycle times and hence the cell production rate can be reduced to the measurement of two parameters :

(i) the growth fraction
(ii) the mean cycle time of proliferating cells.

Thus slow tumour growth could be the result of a long cell cycle time with a large growth fraction, or a short cell cycle time coupled with a small growth fraction. The first problem in the study of cell proliferation in tumours is to be able to decide between these two alternatives.

As regards cell proliferation in human tumours, the experimental difficulties are very great. However, in the light of a detailed knowledge of the situation in experimental tumours it may be possible to plan simple investigations yielding the maximum amount of information. Apart from mitotic index, the only parameter of cell proliferation which has so far been determined in human material is a thymidine labelling index. Occasionally this has been obtained by *in vivo* labelling (Johnson, Rubini, Cronkite and Bond, 1960 ; Clarkson, Ota and Karnofsky,

1962) but more commonly *in vitro* labelling techniques have been used (Johnson and Bond, 1961 ; Wolberg and Brown, 1962 ; Oehlert, Dörmer and Lesch, 1963 ; Reid, 1964 ; Titus and Shorter, 1965 ; Steel and Bensted, 1965). Despite the considerable amount of work which has been done in this direction, little attempt has been made quantitatively to interpret thymidine labelling index. There are difficulties in this, but a possible approach has been suggested by Steel and Bensted (1965) who examined the relationship between labelling index and tumour doubling time. A relationship can be demonstrated theoretically for certain model cell populations and if a sufficient variety of tumours can be found in which both doubling time and labelling index are measurable then experimental confirmation should be possible. The value of such a relationship, if it exists, is that in the case of human tumours, measurements of tumour doubling time can be made on a wide variety of inaccessible tumours and in the case of experimental tumours it becomes possible to examine the growth rate of microcarcinomas.

### CONCLUSIONS

It is difficult to avoid the conclusion that much of the discussion in the literature of the exponential growth of human tumours and the duration of the " silent interval " has been based on insufficient evidence. It may well be that under certain circumstances the exponential extrapolation of a growth curve does yield valid results. However, the present examination of the literature has indicated that this need not be the case, but that errors of a factor of two or more in the estimate of silent interval may be expected.

Other aspects of the growth of human tumours are more deserving of the attention of investigators. The remarkably slow growth of many neoplasms is yet to be explained. Is this inherent in the cell proliferation of well-nourished tumour tissue or is it the result of restrictions imposed by the vascular system of the tumour? It would be well if the emphasis on the silent interval should lead to detailed experimental studies of the growth of microscopic tumours and a fuller understanding of the factors which determine the time between induction and the onset of symptoms. It is through an attack on such problems as these that we may hope to gain some control over the growth rate of human tumours, perhaps even to persuade some of them to grow more slowly and hence to make them less unpleasant to live with.

### SUMMARY

Published literature on the growth rate of tumours in man is reviewed with particular reference to work which gives significant information on the form of human tumour growth curves. Only in tumours of the lung is the data sufficiently good to make any deductions about the equation of growth ; in this situation the growth curves seem to be predominantly exponential.

The widespread practice of extrapolating assumed growth curves back to deduce the time of induction is examined. It is shown that slight deviations from exponential might produce errors of a factor of two or more in the predicted length of the preclinical period. In addition the unpredictable effect of tumour progression complicates the situation still further. The need is for experimental evidence on the growth rate of microscopic tumours.

The occurrence of slow exponential growth in large lung metastases could either be the result of an inherent property of well-vascularised tumour tissue or the statistical result of tumour growth being restricted, probably under vascular limitations. The importance of studies of cell proliferation in deciding between these two hypotheses is stressed.

### REFERENCES

Bertalanffy, F. D.—(1963) *Nature, Lond.*, **198**, 496.
Bertalanffy, F. D. and Lau, C.—(1962) *Cancer Res.*, **22**, 627.
Boag, J. W.—(1949) *Jl R. statist. Soc.* Series B., **11**, 15.
Breur, K.—(1965) Thesis, University of Leiden.
Cairnie, A. B., Lamerton, L. F. and Steel, G. G.—(1965) *Expl Cell Res.* **39**, 528, 539.
Clarkson, B. D., Ota, K. and Karnofsky, D. A.—(1962) *Proc. Am. Ass. Cancer Res.*, **3**, 311.
Collins, V. P.—(1962) *Cancer, N.Y.*, **15**, 387.
Collins, V. P., Loeffler, R. K. and Tivey, H.—(1956) *Am. J. Roentg.*, **76**, 988.
Edwards, J. L., Koch, A. L., Youcis, P., Freese, H. L., Laite, M. B. and Donaldson, J. T.—(1960) *J. biophys. biochem. Cytol.*, **7**, 273.
Foulds, L.—(1956) *J. natn. Cancer Inst.*, **17**, 701.
Garland, L. H., Coulson, W. and Wollin, E.—(1963) *Cancer, N.Y.*, **16**, 694.
Ingleby, H., Moore, L. and Gershon-Cohen, J.—(1960) ' Comparative Anatomy, Pathology and Roentgenology of the Breast '. University of Pennsylvania Press
Johnson, H. A. and Bond, V. P.—(1961) *Cancer, N.Y.*, **14**, 639.
Johnson, H. A., Rubini, J. R., Cronkite, E. P. and Bond, V. P.—(1960) *Lab. Invest.*, **9**, 460.
Laird, A. K.—(1964) *Br. J. Cancer*, **18**, 490.—(1965) *Ibid.*, **19**, 278.
Mendelsohn, M. L.—(1960) *J. natn. Cancer Inst.*, **25**, 485.—(1962) *J. natn. Cancer Inst.*, **28**, 1015.—(1963) ' Cell Proliferation', edited by Lamerton, L. F. and Fry, R. J. M. Oxford (Blackwell).
Mendelsohn, M. L. Dohan, F. C. and Moore, H. A.—(1960) *J. natn. Cancer Inst.*, **25**, 477.
Oehlert, W., Dörmer, P. and Lesch, R.—(1963) *Beitr. path. Anat.*, **128**, 468.
Reid, B. L.—(1964) *J. natn. Cancer Inst.*, **32**, 1059.
Schwartz, M.—(1961) *Cancer, N.Y.*, **14**, 1272.
Spratt, J. S.—(1965) *Cancer N.Y.*, **18**, 14.
Spratt, J. S., Spjut, H. J. and Roper, C. L.—(1963) *Cancer N.Y.*, **16**, 687.
Spratt, J. S. and Spratt, T. L.—(1964) *Ann. Surg.*, **159**, 161.
Spratt, J. S., Ter-Pogossian, M. and Long, R. T. L.—(1963) *Archs Surg., Chicago*, **86**, 283.
Steel, G. G. and Bensted, J. P. M.—(1965) *Eur. J. Cancer* **1**, 275.
Thomlinson, R. H. and Gray, L. H.—(1955) *Br. J. Cancer*, **9**, 539.
Titus, J. L. and Shorter, R. G.—(1965) *Archs Path.*, **79**, 324.
Welin, S., Youker, J. and Spratt, J. S.—(1963) *Am. J. Roentg.*, **90**, 673.
Wimber, D. E.—(1963) ' Cell Proliferation ', edited by Lamerton, L. F. and Fry, R. J. M. Oxford (Blackwell).
Wolberg, W. H. and Brown, R. R.—(1962) *Cancer Res.*, **22**, 1113.